# The proof is in the pudding: patient engagement in studying cannabidiol in mild cognitive impairment

**DOI:** 10.1186/s12906-025-04753-w

**Published:** 2025-01-22

**Authors:** Antonia Keck, Julia-Sophia Scheuermann, Petra Scheerbaum, Elmar Graessel, Kirsten R. Müller-Vahl

**Affiliations:** 1 Center for Health Services Research in Medicine, Department of Psychiatry and Psychotherapy, Uniklinikum Erlangen, Friedrich-Alexander-Universität Erlangen-Nürnberg, (FAU), Erlangen, Germany; 2 Clinic of Psychiatry, Social Psychiatry and Psychotherapy, Hannover Medical School, Hannover, Germany

**Keywords:** Mild cognitive impairment, Alzheimer’s disease, Cannabidiol, Cannabis-based medicine, Patient engagement, Patient and public involvement

## Abstract

**Background:**

Patient engagement (PE) in clinical trials has gained importance yet remains uncommon, particularly in patients with mild cognitive impairment (MCI), a critical precursor to Alzheimer’s disease (AD). Cannabidiol (CBD) shows potential in slowing MCI progression due to its neuroprotective and anti-inflammatory properties. In CBD research, PE is underutilized too. To design a study on CBD for MCI, we administered an online survey to individuals with MCI to better understand their preferences for trial setup and outcomes.

**Methods:**

We asked 209 individuals with MCI to complete an online survey assessing (i) willingness to participate in a trial using CBD; (ii) importance of improvements in various domains; (iii) acceptance of adverse events (AEs); (iv) reasons for AE-related dropout; (v) willingness to undergo blood sampling and lumbar puncture to assess AD pathology; and (vi) willingness to participate in a trial with a 50% chance of receiving a placebo. Data were analyzed with descriptive statistics.

**Results:**

*N* = 118 agreed to participate and *N* = 88 completed the survey. Participants prioritized improvement in cognitive abilities (87.5%), followed by quality of life (63.6%), daily activities (55.7%), sleep (55.7%), pain (52.3%), mood (52.3%), behavior (48.9%), and anxiety (43.2%). Headache (55.7%) was the least accepted AE followed by nausea (46.6%), fatigue (35.2%), and diarrhea (35.2%). Persistent diarrhea (90.9%) and severe fatigue (84.1%) were the main reasons for potential dropout. While most would undergo blood sampling (67.0%), only a minority (21.6%) would accept lumbar puncture. One-third were ready to participate (34.1%), while 54.5% were interested pending details. Among those in favor of participation, 71.6% would participate even with a 50% chance of placebo.

**Conclusions:**

Our study identified cognitive improvement as highly relevant for patients, indicating cognitive assessment tools as primary endpoints in MCI research. Given concerns about AEs, dose titration should be carefully considered to enhance acceptance and prevent AEs. Blood sampling seems well-accepted for AD biomarker assessment. Despite potential AEs, participation in a trial using CBD for MCI is seen as attractive, even under placebo-controlled conditions. This cross-sectional study emphasizes the importance of patient engagement in designing high-quality trials for using CBD to treat MCI.

**Supplementary Information:**

The online version contains supplementary material available at 10.1186/s12906-025-04753-w.

## Background

Active patient engagement (PE) in which patients function as genuine partners in clinical trials – particularly in drug development – has gained enormous importance in recent years. Currently, PE is considered the gold standard in all phases of study design due to the growing awareness of the discrepancy between patient needs and research practice [1]. It is generally accepted that joint action between patient partners and academic researchers is crucial for enhancing the focus on patient-centered healthcare [1]. Zvonareva et al. [2] proposed a framework for conceptualizing the varying degrees of PE in drug research delineating four levels: consultation, involvement, collaboration, and patient-controlled. However, PE remains uncommon in the context of drug research trials. While a variety of disease areas are encompassed by trials that incorporate PE activities, many disease areas remain devoid of such activities in the context of drug research [2]. By actively engaging patient partners in determining how to conduct trials, it is more probable that unmet clinical needs will be addressed and that participant recruitment and adherence will be facilitated.

To the best of our knowledge, PE has not yet been applied in the design of a randomized controlled trial (RCT) investigating the effectiveness and safety of a compound in individuals with mild cognitive impairment (MCI). Mild cognitive impairment is defined as a decline in cognitive abilities that is significantly below what would be expected for an individual’s age and level of education. Frequently regarded as an intermediated phase between normal cognitive aging and dementia, MCI differs from dementia in that it is not accompanied by substantial limitations in activities of daily living (ADL) [3]. Nevertheless, MCI represents a notable alteration in health status and is associated with an elevated risk of progression to dementia with a progression rate of approximately 70% within five years [4]. Moreover, MCI is frequently associated with Alzheimer’s disease (AD), with 40–60% of individuals aged 58 and above diagnosed with MCI also exhibiting underlying AD pathology [5, 6]. A meta-analysis conducted by Jansen and colleagues [7] indicates that the prevalence of AD pathology is 20–30% higher in individuals with MCI compared to those with non-impaired cognition or subjective cognitive impairment, supporting the notion that MCI constitutes a risk condition for AD.

In many cases, direct PE is no longer feasible after the disease progresses and transitions into AD because medical decisions have to be made by patients’ legal representatives on behalf of the individual at this stage [8]. In contrast, people with MCI retain full legal competence. In light of the significant challenges posed by AD to both individuals and society [9], an evidence-based pharmacological treatment for MCI is urgently needed but is currently unavailable [10]. The fact that MCI is easy to diagnose provides the unique chance to implement a treatment that could potentially delay or reduce the probability of progression to AD.

The cannabinoid known as cannabidiol (CBD) has been identified as a promising candidate for slowing down the progression of MCI and therefore delaying the transition to AD due to its neuroprotective and anti-inflammatory properties [11]. In addition, CBD is safe and generally well-tolerated, largely due to its lack of untoward psychoactive sequelae. To date, only a limited number of studies have actively incorporated PE when examining the efficacy and safety of cannabis-based medicines across various indications [12]. Remarkably, approval of highly purified CBD (Epidiolex^®^) to treat seizures in two rare forms of epilepsy (Lennox-Gastaut syndrome and Dravet syndrome) in 2018 was significantly initiated by the fact that the mother of Charlotte, a little girl with Dravet syndrome, had self-initiated adjunctive therapy with a high concentration of CBD from the tetrahydrocannabinol (THC) strain of cannabis to reduce the frequency of Charlotte’s seizures [13]. This case study impressively illustrates the benefits of engaging patients and their relatives or caregivers in initiating new pharmacological therapy strategies.

As part of our planned RCT investigating the effectiveness and safety of CBD in individuals with MCI, we aimed to directly involve patient partners in all steps of the study. To this end, we administered an online survey to individuals diagnosed with MCI who had participated in a recent trial at our center [14]. With this approach, we aimed to design an RCT that employs the most pertinent outcomes for participants, offers a more convenient approach to adverse events (AEs), and considers practical aspects such as acceptance of additional and invasive investigations.

Here, we present the results of the first online survey administered to people with MCI exploring their views on matters of convenience and their preferences regarding the setup of a clinical trial.

## Methods

### Study design and participants

We contacted *N* = 209 people who had previously been diagnosed with MCI at our center and who had recently participated in a non-pharmacological trial (for further information, see Scheerbaum et al. [14]) via email in December 2023 and asked them to participate in the online survey. Of these, *N* = 118 agreed to participate and – after giving written informed consent – were sent a link to the online survey (SoSci Survey; version 3.5.01; [15]). Out of these *N* = 118 people, *N* = 103 opened the survey, and *N* = 88 completed it. To reduce participants’ burden and the time they needed to invest in the study and because collecting participants’ characteristics was not the main focus of this study, we did not collect data on patients’ characteristics in the current study, since these data were available in the study we had recently conducted [14]. However, since the data were collected anonymously, it was not possible to identify the *N* = 88 individuals who participated in this study out of the sample of *N* = 118 who had agreed to participate. In order to provide sample characteristics at least by approximation, we present the participant characteristics of the *N* = 118 individuals who had initially agreed to participate and who were sent the survey link. This information refers to the baseline data in Scheerbaum et al. [14]. Table 1 presents the sample characteristics.

**Table 1:** Characteristics of people with MCI who agreed to participate (*N* = 118) as approximation for actual participants (*N* = 88)

Variable			*N*	Descriptive data
Age: years, *M*^*a*^ (*SD*^*b*^)			118	70.5 (6.6)
Sex: female, *n* (%)			118	63 (53.4)
MoCA^c^ sum score, *M* (*SD*)			118	22.4 (1.6)
B-ADL^d^ sum score, *M* (*SD*)			115^i^	1.6 (0.6)
Education level (ISCED^e^)				
low *n* (%)			118	4 (3.4)
medium *n* (%)			118	41 (34.7)
high *n* (%)			118	73 (61.9)
Household income per month: Euro, *n* (*%*)			118	
500 - < 1,000				3 (2.5)
1,000 - < 1,500				6 (5.1)
1,500 - < 2,000				13 (11.0)
2,000 - < 2,500				19 (16.1)
2,500 - < 3,500				23 (19.5)
3,000 and more				50 (42.4)
not specified				4 (3.4)
Employed: yes, *n* (%)			118	27 (22.9)
Depressiveness: PHQ-9^f^ score, *M* (*SD*)			118	3.9 (2.9)
Vascular risk:				
sum score^g^, *M* (*SD*)			116^i^	1.1 (0.9)
smoking: yes, *n* (%)			118	4 (3.4)
hypertension: yes, *n* (%)			118	51 (43.2)
hypercholesterolaemia: yes, *n* (%)			118	59 (50.0)
diabetes: yes, *n* (%)			116^i^	16 (13.6)
BMI^h^: *M* (*SD*)			118	25.6 (4.6)

Note.^a^*M* = Mean; ^b^*SD* = Standard Deviation; ^c^MoCA = Montreal Cognitive Assessment; ^d^B-ADL = Bayer Activities of Daily Living Scale; ^e^ISCED = International Standard Classification of Education; ^f^PHQ-9 = Patient Health Questionnaire 9; ^g^Number of vascular risk factors: range 0 (no vascular risk factor) to 4 (four vascular risk factors); ^h^BMI = Body Mass Index, normal Range: 18.5–24.9; ^i^There were missing data on B-ADL (3 cases), vascular risk sum score, and diabetes (2 cases each) considered to be missing completely at random (MCAR)

### Online survey

The survey was specially designed for our planned trial “BrainFit-CBD” (currently in the application process) and has not been previously published elsewhere. Participants were informed about the purpose of the survey. In detail, they received the information that the planned trial “BrainFit-CBD” was designed to investigate the effectiveness and safety of CBD in people with MCI. Furthermore, participants were explicitly informed that the purpose of the survey was to learn more about the needs, wishes, concerns, and suggestions of people with MCI in order to be able to take these results into account while designing the study further. In total, the survey comprised 14 questions. It took participants an average of 7.72 min (*SD* = 2.98) to complete the survey. Before the survey was launched, we asked two people with MCI to evaluate the survey’s clarity, comprehensibility, and completeness via an online video interview conducted by one of the authors (AK).

The survey contained the following topics (for the full English version of the survey, please consult additional file 1): (i) importance of improvements (1 = not important to 5 = very important) in various domains including cognitive abilities, activities of daily living, mood, anxiety, behavior, pain, sleep, and quality of life, (ii) acceptance of AEs from CBD (1 = fully acceptable to 5 = not at all acceptable) including the most common AEs from CBD (diarrhea, nausea, fatigue, and headache); (iii) reasons for dropout due to AEs, including persistent diarrhea, mild fatigue, severe fatigue, and mild headache (yes/no); (iv) willingness to undergo blood sampling and lumbar puncture to assess AD pathology (yes; basically yes, but more information is needed; no), and (v) general willingness to participate in a trial investigating the effectiveness and safety of CBD in MCI (yes; basically yes, but more information is needed; no), and more specifically, willingness to participate in an RCT with a 50% chance of receiving a placebo (yes/no).

### Statistics

Data were analyzed with descriptive statistics (frequencies, ranges, percentage, means, and standard deviations), calculated with SPSS version 28.

### Provision of supplementary information for participants

To align with the concept of patient engagement, we empowered participants by providing them with supplementary information on CBD in lay language and expressed our appreciation for their participation by sending a thank you email following the administration of the survey.

### Ethical considerations

The procedures for our recent trial [14], which included sending voluntary follow-up surveys to the study participants, were approved by the Ethics Committee of the Medical Faculty from the Friedrich-Alexander-Universität Erlangen-Nürnberg (Ref. 21-318-1-B).

## Results

### Patients’ characteristics

The sample of *N* = 118 (mean age = 70.6 (+ 6.6) years, *N* = 63 (53.4%) women) – who had agreed to participate – had a mean Montreal Cognitive Assessment score of 22.4 (*SD* = 1.6), indicating MCI [16]. The majority of these people (61.9%, *n* = 73) had a high level of education, according to the International Standard Classification of Education [17]. Almost one quarter (22.9%, *n* = 27) were employed. The mean Patient Health Questionnaire-9 [18] score was 3.9 (*SD* = 2.9), indicating the absence of depression. On average, people had normal body weight with a Body Mass Index of 25.6 (*SD* = 4.6) and had a low level of vascular risk (*M* = 1.1, *SD* = 0.9) calculated as the sum of present vascular risk factors, namely, smoking, hypertension, hypercholesterolaemia, and diabetes. For additional details, see Table 1.

### Online survey

The following results refer to data provided by the *N* = 88 participants who completed the survey. For all the clinical outcomes we assessed, the majority of participants indicated that each outcome was “very important” to them. However, improvement in cognitive abilities was rated as the most important treatment goal (by 87.5% of participants, *n* = 77) followed by (in descending order) quality of life (63.6%, *n* = 56), activities of daily living (55.7%, *n* = 49), sleep (55.7%, *n* = 49), pain (52.3%, *n* = 46), mood (52.3%, *n* = 46), behavior (48.9%, *n* = 43), and anxiety (43.2%, *n* = 38). Table 2 presents the detailed importance ratings of the patient-relevant outcomes.

**Table 2:** Relevance of clinical outcomes as evaluated by participants (*N* = 88)

Variable				Frequencies, *n* (%)				
Importance of improvements^a^				Clinical outcome				
	Cognitive abilites	QoL^b^	ADL^c^	Sleep	Pain	Mood	Behavior	Anxiety
1 (not important)	0 (0)	1 (1.1)	4 (4.5)	5 (5.7)	6 (6.8)	5 (5.7)	6 (6.8)	9 (10.2)
2	1 (1.1)	4 (4.5)	10 (11.4)	7 (8.0)	8 (9.1)	9 (10.2)	7 (8.0)	12 (13.6)
3	2 (2.3)	8 (9.1)	8 (9.1)	10 (11.4)	15 (17.0)	8 (9.1)	17 (19.3)	10 (11.4)
4	8 (9.1)	19 (21.6)	17 (19.3)	17 (19.3)	13 (14.8)	20 (22.7)	15 (17.0)	19 (21.6)
5 (very important)	77 (87.5)	56 (63.6)	49 (55.7)	49 (55.7)	46 (52.3)	46 (52.3)	43 (48.9)	38 (43.2)

Note.^a^Participants were to rate from 1 = *not important* to 5 = *very important*; ^b^QoL = Quality of Life; ^c^ADL = Activities of Daily Living

As the least acceptable AE, 55.7% (*n* = 49) of all participants rated headache as “not at all acceptable”, followed by nausea (46.6%, *n* = 41), fatigue (35.2%, *n* = 31), and diarrhea (35.2%, *n* = 31). Persistent diarrhea was rated as the AE that would most frequently lead to study dropout (indicated by 90.9%, *n* = 80), followed by severe fatigue (84.1%, *n* = 74), mild headache (37.5%, *n* = 33), and mild fatigue (15.9%, *n* = 14). Table 3 presents the acceptance of AEs and probable study dropout due to non-tolerance of AEs as evaluated by participants.

**Table 3:** Non-acceptance of adverse events and frequency of probable discontinuation of the study depending on adverse events of cannabidiol as evaluated by participants (*N* = 88)

Variable		Frequencies, *n* (%)		
Acceptability of side effects^a^		Adverse event		
	Headache	Nausea	Fatigue	Diarrhea
1 (fully acceptable)	1 (1.1)	1 (1.1)	2 (2.3)	2 (2.3)
2	3 (3.4)	2 (2.3)	9 (10.2)	5 (5.7)
3	9 (10.2)	12 (13.6)	19 (21.6)	26 (29.5)
4	26 (29.5)	32 (36.4)	27 (30.7)	24 (27.3)
5 (not at all acceptable)	49 (55.7)	41 (46.6)	31 (35.2)	31 (35.2)
**Reason for study dropout**		**Adverse event**		
	**Persistent diarrhea**	**Mild fatigue**	**Severe fatigue**	**Mild headache**
Yes	80 (90.9)	14 (15.9)	74 (84.1)	33 (37.5)

Note.^a^Participants were to rate from 1 = *fully acceptable* to 5 = *not at all acceptable*

With respect to additional study investigations, 67.0% of participants (*n* = 59) stated that they would be willing to undergo blood sampling to assess AD pathology, while only 21.6% (*n* = 19) agreed to accept a lumbar puncture for this purpose. Additionally, 31.8% (*n* = 28) and 37.5% (*n* = 33) of participants, respectively, indicated that they might be willing to accept these additional investigations but would need more information. Table 4 displays the frequencies of participants’ willingness to undergo invasive procedures.

**Table 4:** Frequencies of participants’ willingness to undergo invasive procedures and RCT^*a*^ participation (*N* = 88)

Variable	Frequencies, *n* (%)	
	Yes	Basically yes^b^
**Willingness to undergo invasive procedure**		
Blood sampling	59 (67.0)	28 (31.8)
Lumbar puncture	19 (21.6)	33 (37.5)
**Willingness to participate in an RCT**		
Overall	30 (34.1)	47 (54.5)
With 50% placebo chance^c^	63 (71.6)	*-* ^*d*^

Note.^a^Participants were asked whether they were willing to participate in an RCT studying cannabidiol (CBD) in mild cognitive impairment (MCI); ^b^”Basically yes” implied that participants required more information; ^c^Participants who chose one of the two “yes” answers were asked if they would also participate even if the chance of receiving a placebo was 50%; ^d^for this question, that response option was not available

Most of the respondents were in favor of participating in a trial for testing CBD for MCI, as 34.1% (*n* = 30) indicated their willingness to participate and another 54.5% (*n* = 47) stated that they might be willing to participate but would need more information. Of those who chose one of the two “yes” answers (“yes” and “basically yes, but more information is needed”), 71.6% (*n* = 63) stated that they would also participate in the RCT even if the chance of receiving a placebo was 50%. Table 4 presents the frequencies of participants’ willingness to participate in the RCT.

## Discussion

This survey aimed to incorporate PE when setting up an RCT to investigate the effectiveness and safety of CBD in treating MCI. In our sample, people with MCI considered improvement in cognitive abilities to be the most important clinical outcome. This finding is consistent with the ICD-11 diagnostic criteria, which identify cognitive impairment as the central symptom of MCI [19]. To conduct patient-relevant research, our findings support the use of a cognitive assessment tool as the primary endpoint, while also considering additional patient-relevant outcomes – such as quality of life, sleep, and activities of daily living – as key secondary endpoints.

In clinical routine settings, the diagnosis of MCI is made on the basis of the results of clinical assessments such as the Montreal Cognitive Assessment [16] or Mini-Mental-State-Examination [20]. According to German guidelines for the diagnosis of AD [10], it is strongly recommended that either a lumbar puncture for cerebrospinal fluid (CSF) analyses or positron emission tomography (PET) imaging are additionally performed to collect and examine AD biomarkers, including amyloid plaques, neurofibrillary tangles, and neurodegeneration [10]. However, PET imaging is extremely expensive [21], has only limited availability, is time consuming, and is therefore hardly possible in the context of larger RCTs, while lumbar puncture is invasive and may cause side effects [22–24]. We were therefore interested in whether people with MCI would be willing to accept a lumbar puncture as an additional investigation in an RCT using CBD. Non-surprisingly, only one fifth of participants indicated their willingness to undergo CSF analyses and another third of patients asked for further information (although written patient information on lumbar puncture was already included in the survey). By contrast, blood sampling was much more accepted by this group of participants, suggesting that, as an alternative to CSF analyses, plasma levels of AD biomarkers should be used to confirm a diagnosis of MCI, although, until now, promising plasma biomarkers for AD (e.g., phosphorylated tau 217; p-tau217) have still been slightly less sensitive than CSF analyses and PET imaging [25]. Additional advantages of blood sample analyses are generally lower costs and general accessibility [26].

With respect to AEs, people with MCI have indicated persistent diarrhea and severe fatigue as AEs that have led to study drop out. This information is important, as the most common side effects of high dose treatments of CBD are diarrhea, nausea, fatigue, and headache, among others [27–29]. So far, no RCTs have been available to investigate the efficacy and safety of CBD in people with MCI. Accordingly, the optimal dose of CBD is also unknown. Based on effects of CBD in other psychiatric indications, it can be assumed that higher doses may be more effective [30]. However, the results of this survey clearly indicate that, in an RCT, the dose titration of CBD should consider the importance of AEs, such as diarrhea and severe fatigue.

Interestingly, in general, participating in an RCT to test the use of CBD seems to be highly attractive to people with MCI. In addition, our results emphasize the need for involvement and informed decision-making, as more than half of the participants requested additional information before participating in the study. As most of the individuals expressed their willingness to participate even if given a placebo, a placebo-controlled trial for CBD appears to be feasible.

Although our study provided valuable insights into PE for planning a trial to test the use of CBD to treat MCI, it is important to acknowledge the limitations of our study. First, due to the inability to match responses with participants, our sample description included individuals who did not participate in the survey, thus rendering it an inaccurate representation of those who indeed completed it. Second, the survey assessments were hypothetical, making different decisions (e.g., regarding treatment discontinuation due to certain side effects) in an actual study possible. Third, our sample consisted of individuals who had already participated in a non-pharmacological trial for MCI, and so they were familiar with the study procedures. Thus, the sample may have been biased because of their general willingness to participate in a clinical trial, and such willingness may have influenced their responses to some extent.

## Conclusions

In summary, this study is the first to incorporate PE into a trial on the use of CBD for MCI, as there are currently no existing PE activities in this area. It provides valuable insights into the concerns, aims, and needs of people with MCI and offers initial implications for planning and conducting future studies involving this population. Our main message is to encourage the incorporation of PE activities in the drug research process, not only to reflect the goals and needs of patients but also to improve the quality of research.

## Electronic supplementary material

Below is the link to the electronic supplementary material.

Supplementary Material 1

Supplementary Material 2

## Data Availability

The datasets used and/or analysed during the current study are available from the corresponding author on reasonable request.

## Abbreviations

AD: Alzheimer’s disease
AE(s): Adverse event(s)
CBD: Cannabidiol
CSF: Cerebrospinal fluid
M: Arithmetic mean
MCI: Mild cognitive impairment
PE: Patient engagement
PET: Positron emission tomography
RCT: Randomized controlled trial
THC: Tetrahydrocannabinol
