# Supplementary material for: The proof is in the pudding: patient engagement in studying cannabidiol in mild cognitive impairment
Source: BMC Complement Med Ther. 2025 Jan 22;25:19. doi: 10.1186/s12906-025-04753-w (PMC11755935; doi:10.1186/s12906-025-04753-w)
Supplement: Supplementary file 1 — Supplementary Material 1 [file 12906_2025_4753_MOESM1_ESM.pdf]

Survey for planning the new ‘BrainFit-Cannabidiol’ study

Note: The original survey was conducted in German on the online platform SociSurvey (https://www.soscisurvey.de). Therefore, presentation of the survey this file differs from the original online version.

Introduction

Dear Sir or Madam,

Thank you for taking part in our survey. This survey is aimed at previous BF-N study participants. We would like to use the results of the survey to plan our new study according to the wishes, concerns and needs of those affected. By taking part, you therefore have the opportunity to actively help shape the research.

Questions

1. How important is it to you that treatment leads to an improvement in the following areas? (even if you are not currently affected)

Patients were to rate the following areas from 1 = not important to 5 = very importantMental abilities (e.g. short-term memory, ability to concentrate)

   a) Skills related to activities of daily living (e.g. telephoning, laundry)
   b) Mood (e.g. sadness, joylessness, resignation)
   c) Fears (e.g. avoiding contact with others, not daring to leave the house)
   d) Verhaltensauffälligkeiten (z.B. Unruhe, Gereiztheit, Aggressivität)
   e) Pain
   f) Sleep
   g) Quality of Life

2. Would you be willing to take cannabidiol (CBD) extracted from the hemp plant orally (as an oily solution, capsule or oral spray) to treat mild memory impairment?
   a) Yes
   b) Basically yes, but I need more information

c) No

3. How would you rate your knowledge of cannabidiol (CBD)?

   Participants were to rate their knowledge from 1 (“no knowledge”) to 5 (“I know all about it”)

4. How acceptable would the following possible side effects of treatment with cannabidiol (CBD) be four you?

Patients were to rate each of the following side effect from 1 = fully acceptable to 5 = not at all acceptable

a) Diarrhea

b) Nausea

c) Fatigue

d) Headache

5. Which of the following possible side effects of treatment with the drug cannabidiol (CBD) would cause you to stop treatment?

For each side effect, patients were to choose either ‘yes’ or ‘no’.

a) Persistent diarrhea

b) Mild fatigue

c) Severe fatigue

d) Mild headache

6. Blood samples will be taken as part of the planned study. Would you agree to your blood also being analysed for signs of Alzheimer's disease?

   a) Yes

   b) Basically yes, but I need more information

   c) No

7. Would you be willing to have cerebrospinal fluid taken as part of the planned study in order to find evidence of Alzheimer's disease? You can find further information on the collection of cerebrospinal fluid here (https://www.gesundheitsinformation.de/was-passiert-bei-einer-lumbalpunktion.html)

   a) Yes

   b) Basically yes, but I need more information

c) No

8. Can you imagine taking part in a telephone or online visit once a month as part of the planned study?
   a) Yes
   b) No
   c) I don’t know
9. Would you be willing to visit one of the study centres in person twice during the course of the study (after 6 and after 12 months)?
   a) Yes
   b) No
   c) I don’t know
10. What is the maximum distance between the study centre and your place of residence if you had to travel there two to three times within 12 months to participate in the study?
    a) Maximum 20 km
    b) Maximum 50 km
    c) Maximum 100 km
    d) Maximum 200 km
    e) Distance does not matter
11. Where do you obtain information on medically important topics? (Multiple answers possible)
    a) Newspaper
    b) Magazines (e.g. available in pharmacies)
    c) Internet
    d) Television
    e) Radio
    f) Information brochures
    g) General Practitioner
    h) Pharmacist
    i) Other
12. How concerned are you about the possibility of developing Alzheimer’s dementia?

Participants were to rate their concern from 1 (“not concerned at all”) to 5 (“very concerned”)

13. Can you generally imagine taking part in the planned ‘BrainFit-CBD’ study?

    a) Yes
    b) Basically yes, but I need more information
    c) No

Patients who chose one of the two “yes” answers (“yes” and “basically yes, but I need more information”) were redirected to the following additional question:

13.1 Would you also take part in the study even if the chance of receiving a placebo (a drug without a real active ingredient) was 50%?

    a) Yes
    b) No

14. May we contact you again to give you more information about the planned study? If you agree, you can provide your E-Mail address here.

Closing statement

Thank you for taking part in this survey! You are making an important contribution to the planning of the study.
