# Supplementary material for: The proof is in the pudding: patient engagement in studying cannabidiol in mild cognitive impairment
Source: BMC Complement Med Ther. 2025 Jan 22;25:19. doi: 10.1186/s12906-025-04753-w (PMC11755935; doi:10.1186/s12906-025-04753-w)

Figure 1

Relevance of clinical outcomes as evaluated by participants (N = 88)

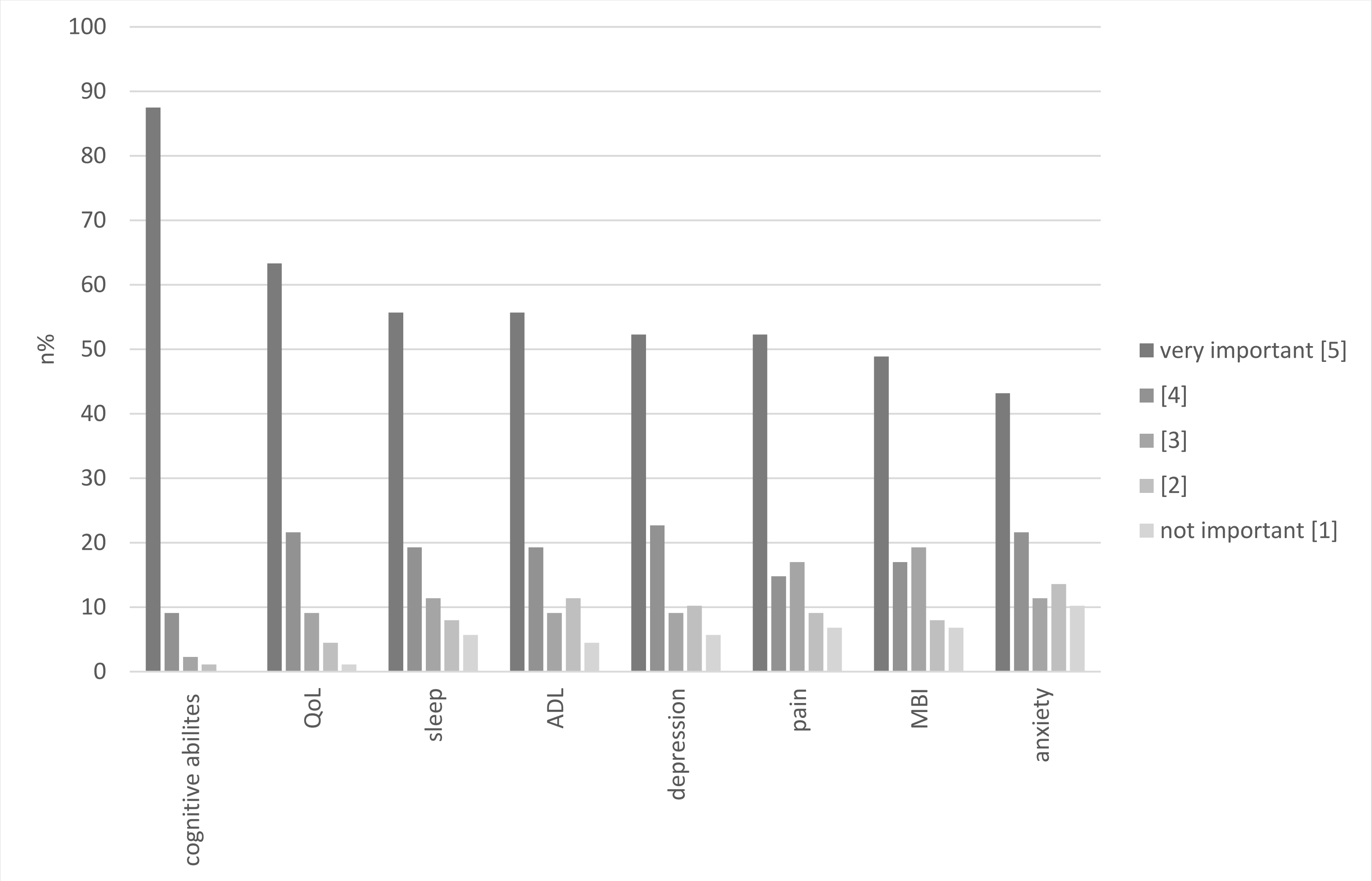

Note. ADL = Activities of daily living; MBI = Mild behavioral impairment; QoL = Quality of life.

Figure 2

Non-acceptance of adverse events of cannabidiol (CBD) as evaluated by participants (N = 88)

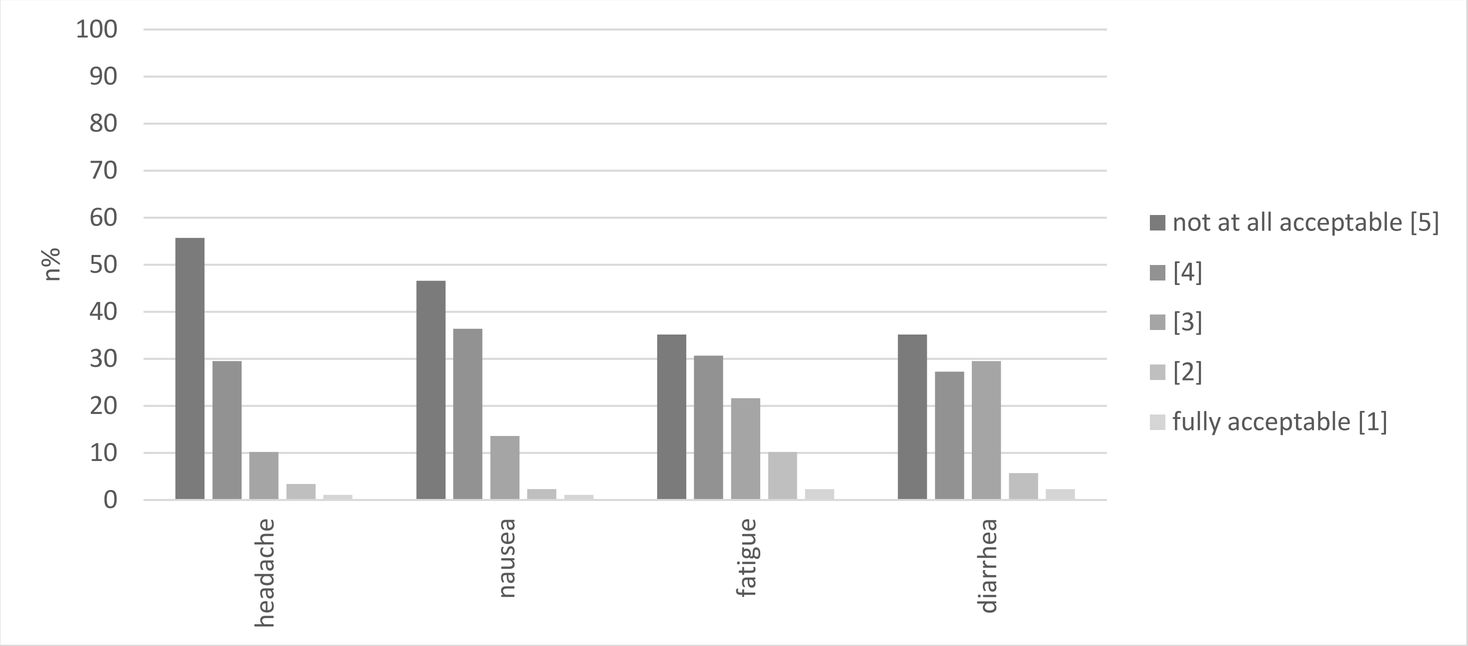

Figure 3

Frequency of probable discontinuation of the study depending on side effects of cannabidiol (CBD) as evaluated by participants (N = 88)

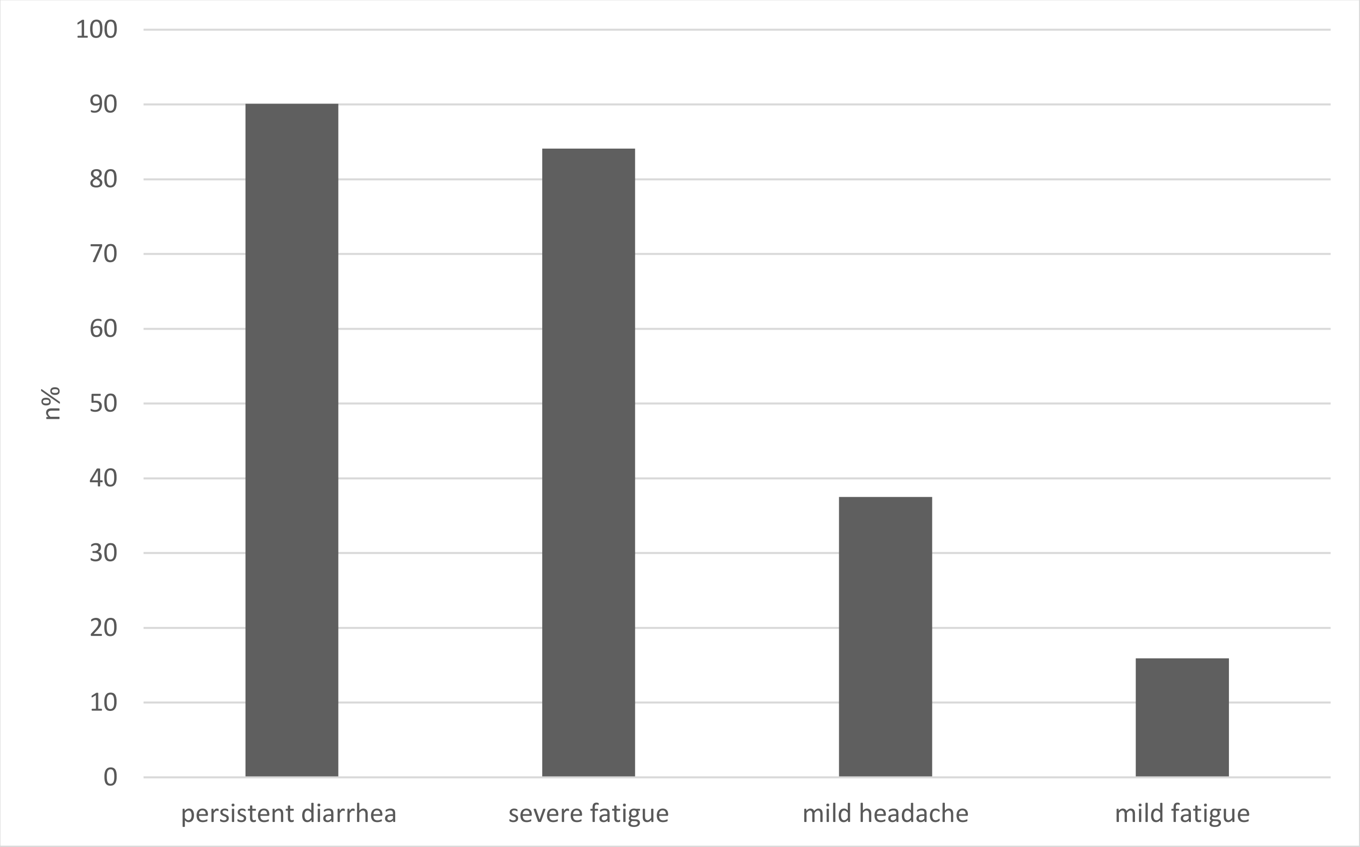

Supplement: Supplementary file 2 — Supplementary Material 2 [file 12906_2025_4753_MOESM2_ESM.pdf]
